# Adaptation of balancing behaviour during continuous perturbations of stance. Supra-postural visual tasks and platform translation frequency modulate adaptation rate

**DOI:** 10.1371/journal.pone.0236702

**Published:** 2020-07-31

**Authors:** Stefania Sozzi, Antonio Nardone, Marco Schieppati

**Affiliations:** 1 Centro Studi Attività Motorie, ICS Maugeri SPA SB, IRCCS, Institute of Pavia, Pavia, Italy; 2 Department of Clinical-Surgical, Diagnostic and Paediatric Sciences, University of Pavia, Pavia, Italy; 3 Neurorehabilitation and Spinal Units, ICS Maugeri SPA SB, IRCCS Institute of Pavia, Pavia, Italy; 4 ICS Maugeri SPA SB, IRCCS, Institute of Pavia, Pavia, Italy; Toronto Rehabilitation Institute - UHN, CANADA

## Abstract

When humans are administered continuous and predictable perturbations of stance, an adaptation period precedes the steady state of balancing behaviour. Little information is available on the modulation of adaptation by vision and perturbation frequency. Moreover, performance of supra-postural tasks may modulate adaptation in as yet unidentified ways. Our purpose was to identify differences in adaptation associated to distinct visual tasks and perturbation frequencies. Twenty non-disabled adult volunteers stood on a platform translating 10 cm in antero-posterior (AP) direction at low (LF, 0.18 Hz) and high frequency (HF, 0.56 Hz) with eyes open (EO) and closed (EC). Additional conditions were reading a text fixed to platform (EO-TP) and reading a text stationary on ground (EO-TG). Peak-to-peak (PP) displacement amplitude and AP position of head and pelvis markers were computed for each of 27 continuous perturbation cycles. The time constant and extent of head and pelvis adaptation and the cross-correlation coefficients between head and pelvis were compared across visual conditions and frequencies. Head and pelvis mean positions in space varied little across conditions and perturbation cycles but the mean head PP displacements changed over time. On average, at LF, the PP displacement of the head and pelvis increased progressively. Adaptation was rapid or ineffective with EO, but slower with EO-TG, EO-TP, EC. At HF, the head PP displacement amplitude decreased progressively with fast adaptation rates, while the pelvis adaptation was not apparent. The results show that visual tasks can modulate the adaptation rate, highlight the effect of the perturbation frequency on adaptation and provide evidence of priority assigned to pelvis stabilization over visual tasks at HF. The effects of perturbation frequency and optic flow and their interaction with other sensory inputs and cognitive tasks on the adaptation strategies should be investigated in impaired individuals and considered in the design of rehabilitation protocols.

## Introduction

Experimental protocols centred on periodic displacements of the support base upon which individuals stand have contributed important information on human balance control [1–4] and have been successfully exploited as rehabilitation procedures for various patient populations with balance disorders [5–7]. During continuous and predictable perturbations, the appropriate balancing behaviour requires a fine coordination of body segments’ motion. This undeniably depends on the visual condition, whereby the head tends to be stabilized in space (‘head-fixed-in-space’) with eyes-open (EO) and good visual acuity, and oscillates largely with poor visual acuity or with eyes-closed (EC) (‘head riding the platform’) [8–12]. Vision is an important factor for head stabilization, and its effect is independent of standing *per se*. For example, when translation of the lower body is constrained in seated individuals, relative trunk motions are smaller under EO- than EC-condition [13], indicating a better performance for upper body stabilization in space with vision.

The postural system responsible for the maintenance of equilibrium under demanding standing conditions, such as displacement of the support base, relies both on the integration of sensory information from the peripheral receptors and on the proactive adjustments when the perturbation is predictable [1, 14]. Time-consuming processes have been described under various conditions requiring integration of diverse sensory inputs (e.g., haptic, proprioceptive, visual) for stance control [15], ranging from modulation of stretch-reflex responses [16] to changes in torques acting on the support base in response to haptic stimuli [17–22] or to modulation of static and dynamic balancing behaviour in response to changes in visual [23–25] and proprioceptive input [26, 27]. During periodic translations of the support base, a certain time interval elapses from the trial onset until a steady-state of head and body oscillation is reached [3, 28–32]. This may account for changes in balance control dynamics depending on explicit predictions of the sequence of perturbation cycles [33].

Balancing with EO on a platform translating in the antero-posterior (AP) direction at high frequency bears virtually no adaptation, the steady state of body segment motion and muscle activity being reached shortly (one-two perturbation cycles) after the start of the sequence of platform translations [32]. The EC condition is instead associated with a slow adaptation process, whereby several mechanical and EMG variables follow a decaying exponential time course [32]. The process of adaptation may contain elements connected with the shift from a ‘safe’ state (standing quietly) to a challenging condition (the movement of the support base), the quality of the sensory feedback and sequence-specific features (the frequency of continuous translations).

The head antero-posterior (AP) displacements have been recently studied under conditions requiring the execution of a visual supra-postural task [34]. That investigation has shown that the ‘head-fixed-in-space’ behaviour with EO can be revoked during the performance of a visual task consisting in reading a text that moves with the platform. The finding has challenged the common view that vision stabilizes the head in space in order for the body to exploit the head as an inertial reference during dynamic tasks [35–39]. Bonnet et al. [40], based on a quiet-standing protocol, suggested that precise visual tasks may require the brain to control synergistic relations between eye and body movements [41, 42] instead of stabilizing head posture. The question of whether the process of adaptation of a *dynamic* postural behaviour depends on the visual condition at hand has received little consideration. Focussing attention on a visual task unrelated to the balancing effort might delay the attainment of the steady state or possibly modify the amplitude of the body adjustments. A connected issue is whether adaptation is linked to the process of head stabilization in space or is present even when the head may not be stabilized as during a supra-postural precision visual task, where priorities would be different.

In the present study, we wanted to quantify the habituation rate of the body AP displacement under conditions that feature distinct visual states, including those implying the performance of supra-postural visual tasks. Indeed, the ‘fixed-in-space’ head behaviour EO becomes a ‘head-riding-the-platform’ behaviour when individuals focus on an object moving jointed with the platform, in spite of full vision availability [34]. In this case, vision helps accomplish the visual task optimally by keeping a constant head-text distance, rather than by producing head and body stabilization in space as a necessary outcome. Hence, a conflict would ensue between the head stabilization in space with EO and the new motion pattern. Further, it is not clear whether body forward or backward inclination is part of the adaptation process and affects the periodic head motion. Moreover, longer durations of the perturbation cycles (lower perturbation frequencies) may affect the adaptation process, potentially slowing the attainment of the steady state compared to short cycle durations. It is not clear whether the process of adaptation entails distinct strategies and time constants producing different rates of convergence to a steady state.

We have investigated the adaptation phenomenon of head displacement under both low frequency (LF) and high frequency (HF) platform translations, with EO and EC, and while reading a text. In turn, the text was secured to the mobile platform itself and moved jointly with it (EO-TP) or was fixed to the ground, immobile in space (EO-TG). The study aimed to 1. confirm in young individuals the fast adaptation at HF of the head peak-to-peak (PP) displacement with EO compared to the gradual adaptation with EC, 2. evaluate the different features of the adaptation pattern at LF and HF platform translations, including body orientation in space and coordination between head and pelvis motion, and 3. establish whether the adaptation differs between the condition EO (without an associated visual task) and the condition EO with a supra-postural visual task. Identifying a different adaptation process (rate and amplitude) of head and pelvis motion during the performance of the supra-postural visual tasks would help understand the mechanisms driving the balance adaptation, the relevance of head stabilization in the maintenance of the dynamic equilibrium, the interference of a cognitive task on a dynamic balancing behaviour, and possible deficits in aged populations or in people with equilibrium disorders.

## Methods

### Participants

Twenty healthy young adults (10 males and 10 females) participated in the study. Age was 27.7 ± 5 years (mean ± SD), height 173.8 ± 9.2 cm and weight 66 ± 10.7 kg. All participants gave written informed consent to participate in the experiments. These were performed in accordance with the Declaration of Helsinki and approved by the institutional Ethics Committee (Istituti Clinici Scientifici Maugeri, approval number # 2257 CE). These individuals participated in a previously published study on the effects of vision on the balancing behaviour [34]. Here, the data recorded in the same cohort have been analysed further in order to address the effects of visual task and translation frequency on the adaptation of the balancing behaviour across the period of repeated perturbations.

### Tasks and procedures

The methodology has been described in detail in [34]. Here, we briefly report the main procedures and the new analytical methods. Participants were asked to stand with bare feet spaced about 10 cm apart and with the arms by their side on a mobile platform (Officina Lomazzi, Italy). The platform translated in the horizontal plane, in the antero-posterior (AP) direction following a sinusoidal function at 0.18 Hz (low frequency, LF) or 0.56 Hz (high frequency, HF). The amplitude of the AP platform translation was 10 cm for both frequencies, constant across all the trials. Each acquisition epoch consisted of a period (lasting at least 5 s), during which participants stood on the still platform, and of a period of continuous AP platform translations (27 consecutive cycles at LF and 31 at HF). For the statistical analysis we considered the cycles from 1 to 27 for both translation frequencies. Hence, each perturbation period lasted about 3 min at LF and about 1 min at HF. For each frequency, participants performed two trials for each of these four different visual conditions: 1. eyes open (EO) during which they were free to look at the patterned laboratory space, 2. reading a text securely positioned in front of them on a light-weight stand rigidly fastened to the base of the moving platform (EO-TP) so that the text moved jointly with the platform, 3. reading a text fixed to the ground (EO-TG) in front of them, and 4. eyes closed (EC). The text was positioned at eye level under both EO-TP and EO-TG conditions. At the beginning of each trial, participants were free to choose the place of their standing position on the platform in order to have a comfortable view of the text. As a consequence, the distance between head and text (for both EO-TP and EO-TG) varied across participants between 42 cm and 53 cm. The reading-text was printed on a A4 sheet (normally spaced words, 13-point black Arial font, 33 horizontal lines spaced by 1.5 lines, aligned left). Participants began reading the text aloud just before the platform started to move and continued reading until the platform stopped. The content of the text changed from trial to trial. They were aware of the onset of the acquisition but were not warned of the forthcoming onset of the platform movement. For each participant, the trials at the same frequency were performed in sequence (LF before HF) to minimize startle reactions related to unexpected changes in the frequency of the platform translations from one to the following trial [43]. Visual conditions were randomized within frequencies and participants. Trials at different visual conditions and platform translation frequencies were separated by a time-period (from 2 to 5 min) during which participants were free to move and rest. The entire recording session lasted less than 2 hours, including the resting periods.

### Data recording and analysis

The head and pelvis displacements were acquired by means of an optoelectronic device (Smart-D, BTS, Italy) composed of 12 infrared cameras. The sampling frequency was 140 Hz. Eight reflective markers were placed in the following body positions: vertex, forehead, and bilaterally on the lateral part of a replacement hard-hat suspension, and bilaterally on the pelvis (anterior-superior iliac spine), considered a proxy for the centre of mass under the present conditions. A marker was placed on the platform in order to record the platform translations. Another marker was fixed to the text sheet.

For each trial and for each visual and frequency condition, the mean AP position in space and the PP displacement of head and pelvis were measured in each participant, cycle by cycle. The mean AP positions were then referred to the values of the mean AP position during the first cycle. The corresponding data of the two trials performed by all participants in each condition were then averaged and the relationship between AP position and PP displacement amplitude was evaluated by the Spearman’s test and the correlation coefficient (ρ). This non-parametric model, robust in the presence of outliers, has been used to take into account the discrepancies between the values of the initial cycles compared to the following cycles.

The amplitudes of the PP displacement during the successive translation cycles showed in most cases an exponential trend that construed an adaptation behaviour [32, 44, 45]. In order to quantify the adaptation rate, for each participant and for both head and pelvis we fitted the mean PP values over the consecutive cycles with an exponential function (y = A + Be^−C*t^). To this aim, the iterative conjugate gradient method of the Excel® Solver Utility was used, τ(tau) = -1/C being the time-constant of the exponential function, A the value at steady state (asymptote) and A + B the value of the intercept (I) with the ordinate at the first cycle [18]. A, B and C parameters were computed by using the minimum sum squared algorithm. Of note, the time-constant τ was expressed in cycles. It can be transformed into time units by multiplying the cycle number by the cycle duration (i.e., 5.6 s and 1.8 s for LF and HF, respectively). For each trace, the goodness of the exponential fit was estimated by the Pearson’s R coefficient, and the significance of R noted. In order to give a measure of the amplitude of the changes in PP values from the beginning to the end of the perturbation sequence, an adaptation index (AI) was calculated from the asymptote and intercept values as AI = (A-I)/(A+I). A negative value indicated that the PP value was smaller at the end of the perturbation sequence compared to the beginning (i.e., the fitted function had a decaying exponential trend), and vice versa for positive values. For example, the AI is about—0.3 when the asymptote is about half of the intercept value for a decaying exponential function, while a value of AI near zero points to a negligible adaptation extent. The mean values of the AP positions of head and pelvis during the cycles from 15 to 27 for all test conditions were considered to represent the steady state of the balancing behaviour. This was based on the duration of the mean time-constants of the adaptation rate calculated on the PP values (3*τ < 15 cycles, see Results), which allowed to estimate that the adaptation phenomenon was completed by the middle of the perturbation cycles.

The spatio-temporal coordination between the movement of the head and that of the pelvis was studied cycle-by-cycle across participants and visual conditions by the cross-correlation (CC) analysis between the traces of the head and pelvis markers, using a software developed in LabView (National Instruments, Austin, Texas). A positive value of the CC coefficient (R) calculated at time lag = 0 indicates that head and pelvis move in the same direction, a negative value indicates that head and pelvis move in the opposite direction. The values of the CC coefficient over the subsequent cycles were fitted with the same exponential model used for the PP data and analysed by the same procedure. Likewise, the mean value of the CC coefficient calculated for each cycle was plotted against the value of the corresponding head PP displacement amplitude, for each visual condition and translation frequency, and a linear regression model was fit to the data. The coefficient of determination (R^2^) was used to assess the goodness of the linear fit.

### Statistical analysis

The normality and homogeneity of variances were analyzed by the Kolmogorov-Smirnov test and Levene’s test, respectively. Assumptions for parametric statistics were met for all variables of interest. The differences in mean AP positions at steady state compared to their initial mean values were evaluated for both head and pelvis and for all visual and frequency conditions by the paired Student’s t test. A 2 x 2 x 4 repeated-measure ANOVA (factors: LF and HF; head and pelvis segments; EO, EO-TP, EO-TG and EC conditions) was used to compare the mean time-constants and adaptation indices of the exponential functions fitted to the successive PP values. A 2 x 4 repeated-measure ANOVA (factors: frequencies and visual conditions) was used to compare the mean CC coefficient after z-transformation and the mean time-constant of the CC coefficients. A 2 x 2 x 4 repeated-measure ANOVA (head PP and CC coefficient, frequencies, visual conditions) was used to compare the mean time-constant of head PP displacement amplitudes and CC coefficients. When Mauchly’s test indicated that the assumption of sphericity had been violated, the Greenhouse-Geisser correction was applied (the χ^2^ test statistic and the adjustment for sphericity violation (ε) are indicated in the text). For all ANOVAs, the post-hoc test analysis was done with the Tukey’s HSD test. A value of p < 0.05 was considered statistically significant. The mean value of the AI for each body segment, visual and frequency condition has been compared to zero by means of the Student’s t-test, separately per condition, in order to assess the significance of the mean extent of the adaptation of head and pelvis PP displacements. We used the software package Statistica (StatSoft, USA).

## Results

### Head and pelvis Peak-to-Peak (PP) displacement during the continuous platform translations. The effect of visual condition and translation frequency

**Fig 1:**
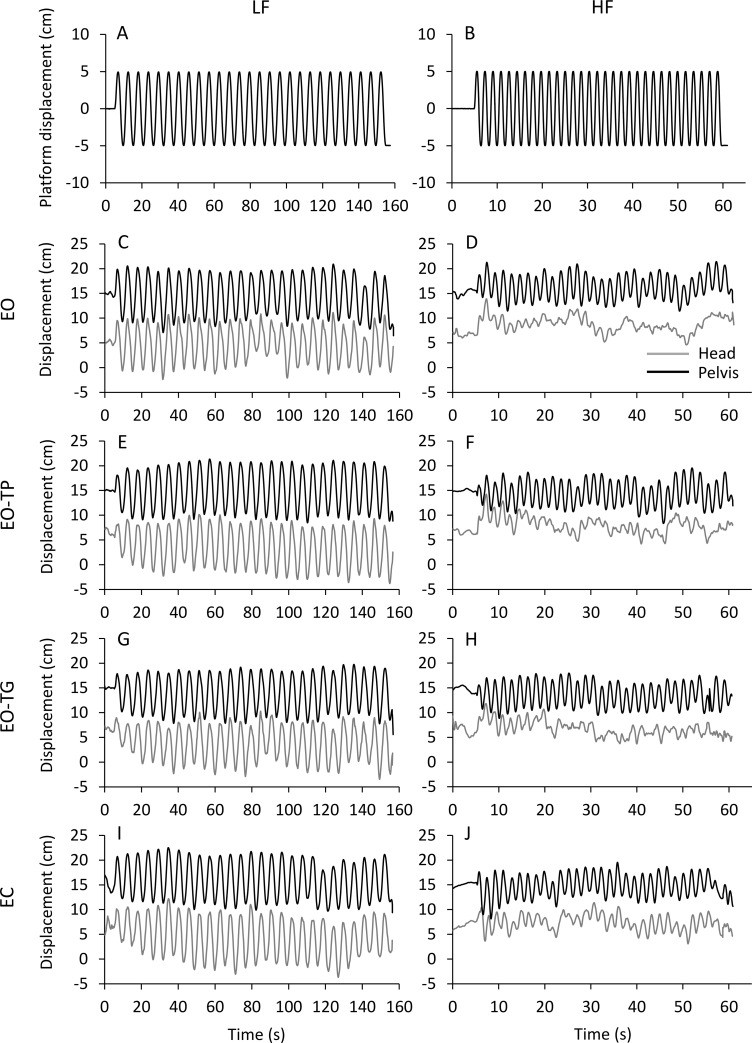
A and B: platform AP translation traces. The platform started to move at t = 5 s and delivered consecutive translation cycles at 0.18 Hz (LF, left panel) and 0.56 Hz (HF, right panel) with a constant PP translation amplitude of 10 cm. C to J: head (grey traces) and pelvis (black traces) AP displacement with eyes open (EO, C and D), reading the text moving with the platform (EO-TP, E and F), reading the text fixed to the ground (EO-TG, G and H) and with eyes closed (EC, I and J). Increasing values on the ordinate indicate forward movement.

Fig 1 shows the traces of the AP displacement of head (grey traces), pelvis (black traces) and platform (top panels) in a representative participant during the balance perturbation trials administered at both low (left) and high (right) frequency under the different visual conditions: from top to bottom, eyes-open no task (EO); eyes-open while reading the text moving with the platform (EO-TP); eyes-open while reading the text fixed to the ground (EO-TG) and eyes-closed (EC). Head and pelvis displacements feature some variability across the entire trial. Overall, the PP displacements are larger at LF than at HF. A steady state is reached after a number of cycles and is maintained until the end of the trial under both frequency conditions. However, there is a clear trend, in particular for the head, to increase and diminish the PP displacement amplitudes at LF and HF, respectively, under all visual conditions.

**Fig 2:**
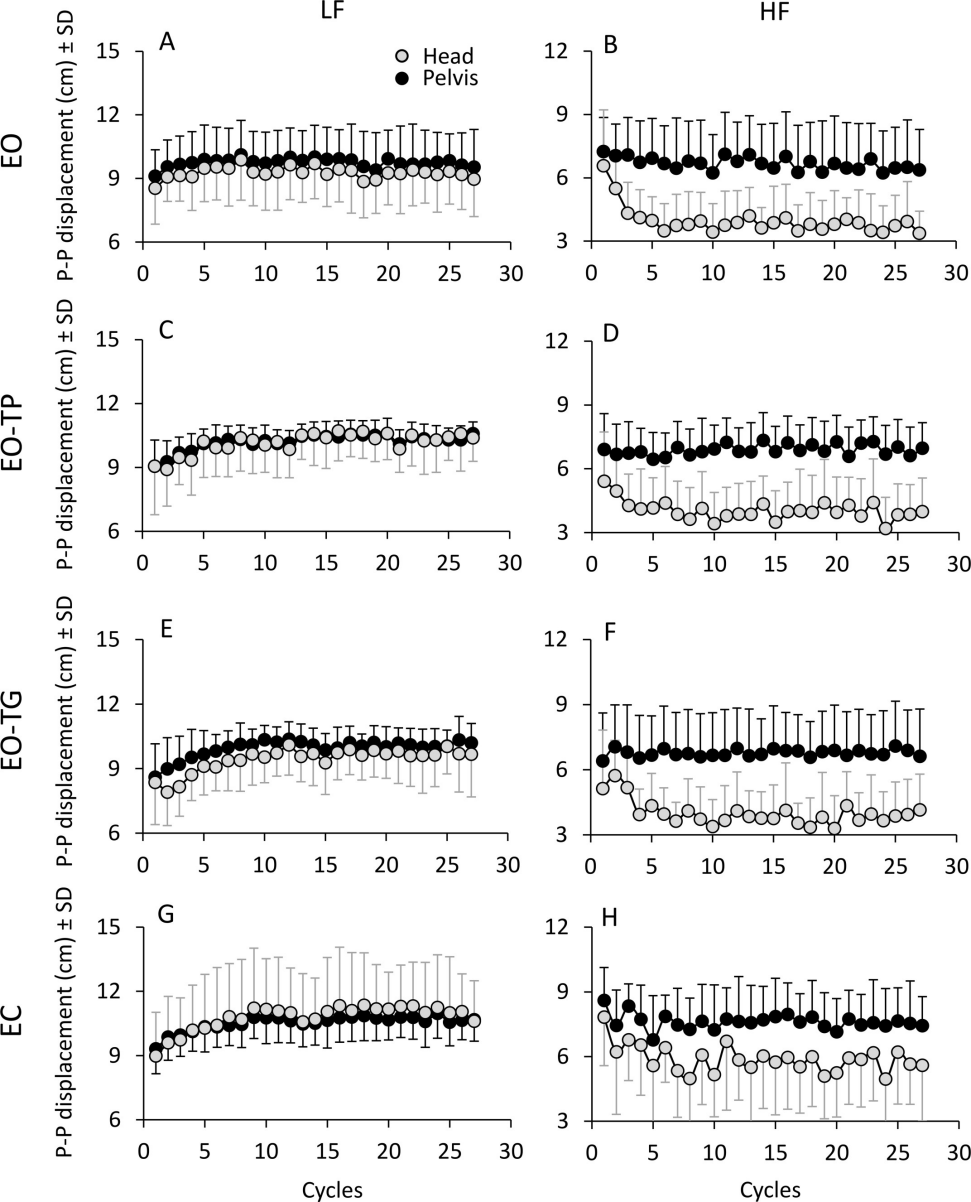
**Mean amplitude of PP displacement calculated cycle by cycle for LF (left column) and HF (right column) perturbations.** Grey and black dots correspond to the head and the pelvis, respectively. For all visual conditions, the amplitudes of head and pelvis PP displacement showed an increasing trend at LF and a decreasing trend at HF. Note the shift in the ordinate values between the LF and HF panels.

Fig 2 shows the mean values of the amplitude of the PP displacements of the body segments during the perturbation period. Overall, at LF (left column), the PP displacement amplitudes of head and pelvis increased in parallel over time and reached the steady state before the middle of the perturbation period. These changes were rapid with EO, and slower under the other visual conditions (EO-TP, EO-TG and EC). At HF (right column), the pelvis maintained its PP displacement amplitude relatively constant across cycles, but there was a remarkable reduction in the head PP displacement amplitudes. With EC, the head PP displacement diminished as well, but less than in the other visual conditions, a sign of reduced relative head stabilization in space. Again, these changes were more rapid with EO than in the other visual conditions.

**Fig 3:**
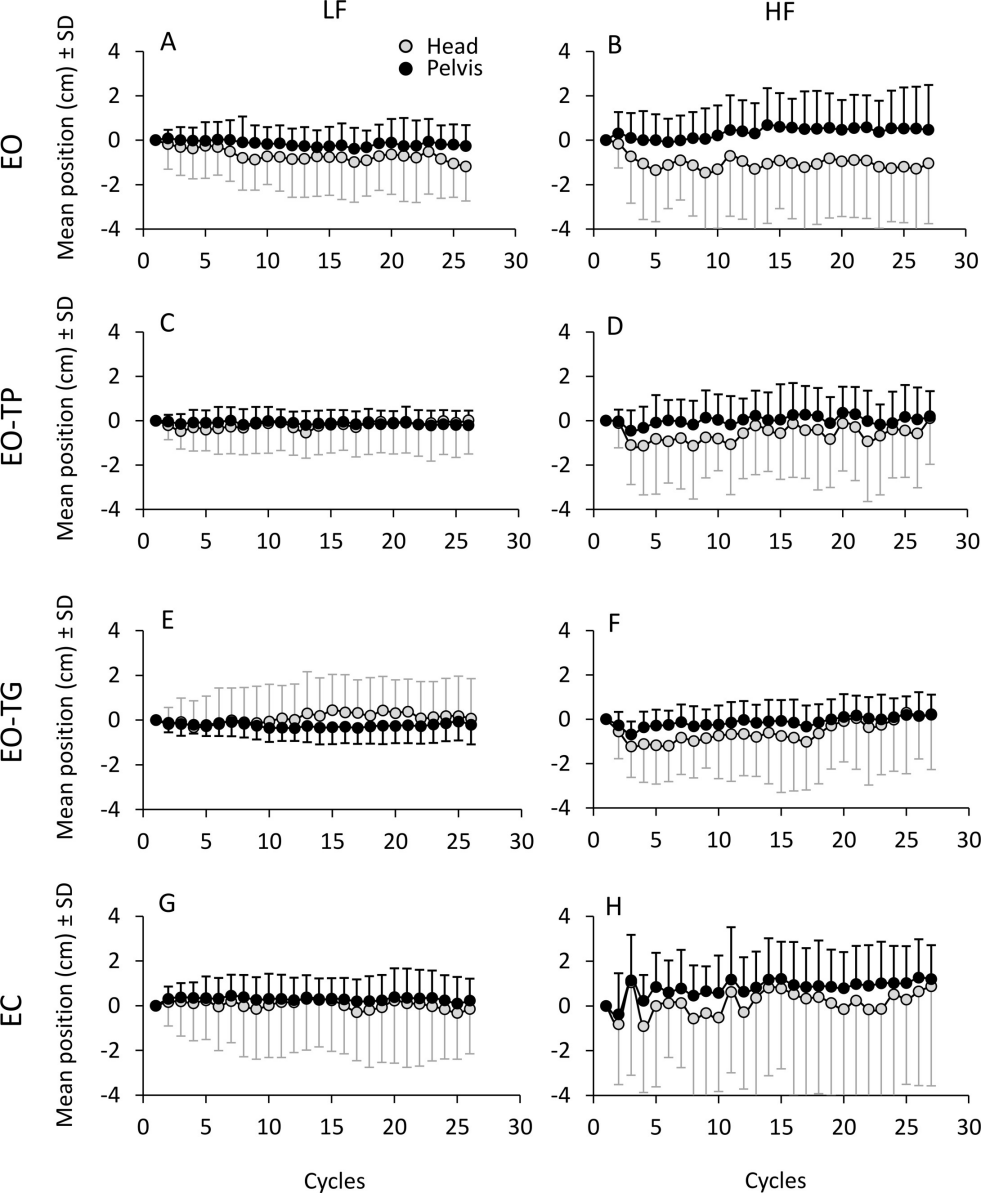
**Mean AP position calculated cycle by cycle for LF (left column) and for HF (right column) under the different visual conditions.** Grey dots refer to the head, black dots to the pelvis. The position calculated in the subsequent cycles are referred to the position of the first cycle (positive is forward). The pelvis mean AP position are fairly constant from the beginning to the end of the perturbation cycles under all visual conditions at LF and move slightly forward at HF.

Fig 3 shows the effect of the successive perturbation cycles on the mean positions of head and pelvis. At LF, the mean positions of both segments were almost superimposed from the beginning to the end of the trials. At HF, the mean positions slightly diverged. The head mean positions (grey circles) were very close to those of the pelvis with EO-TP and EO-TG and were detached in a modest range with EO and EC. There was a tendency to shift forward for both the head (not so with EO) and the pelvis. These shifts did not exceed 1 cm, on the average, with a relatively high inter-individual variability, as attested by the height of the SD bars. Comparison of the mean values of the AP positions at steady-state to the initial positions yielded no differences, regardless of translation frequency, visual condition and body segments (paired t-test, p > 0.1 for all comparisons), except for a slight backward shift of the head with EO, LF and HF (p < 0.05, for both comparisons) and a forward shift of the pelvis with EC at HF (p < 0.01).

**Fig 4:**
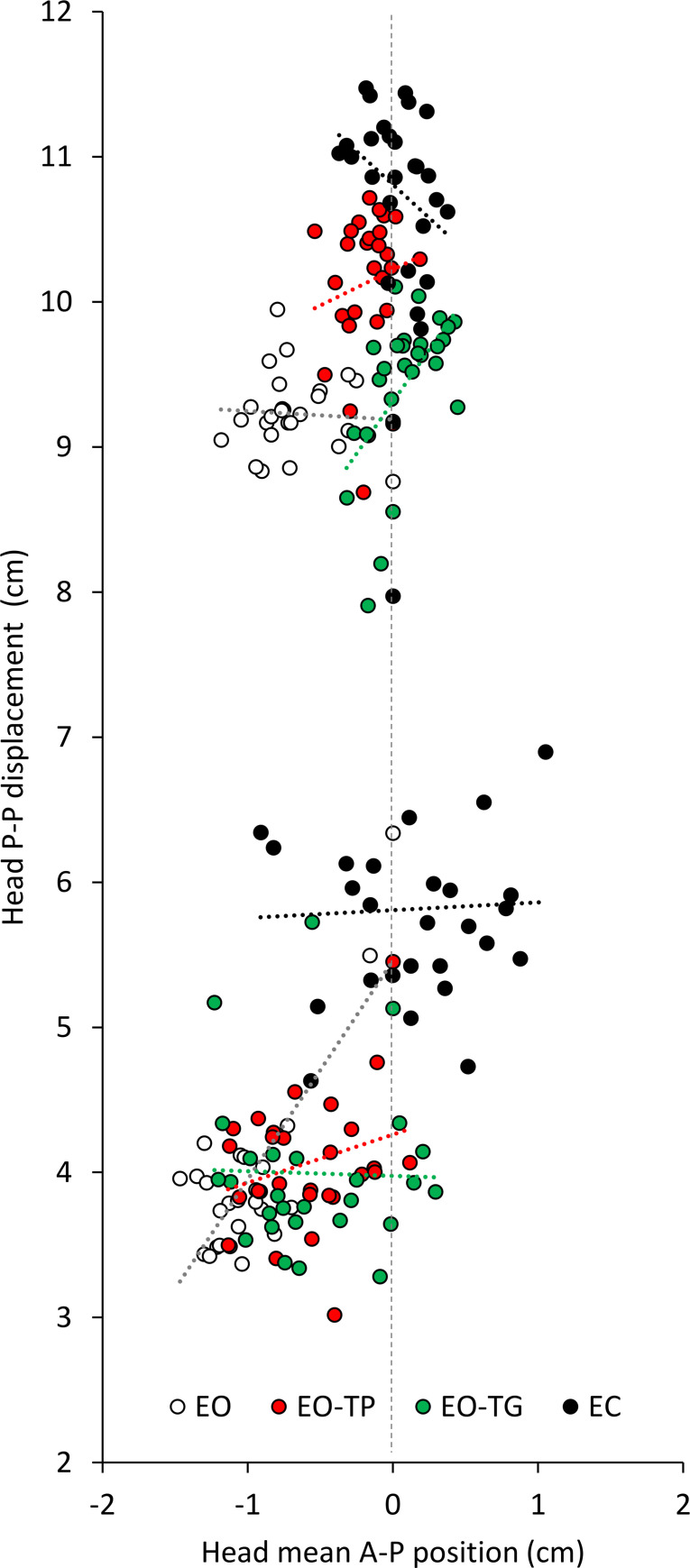
Relationship between head PP displacement and AP position (referred to the initial position). The head mean PP displacement amplitudes (ordinate) are plotted against the head mean AP positions (abscissa) for all the visual conditions and platform translation frequencies. Each symbol (n = 27) corresponds to the mean value of the variables of all participants averaged cycle by cycle. White circles refer to the EO condition, red to EO-TP, green to EO-TG and black circles to EC. Due to the largely different values of head PP displacements at the two frequencies, the data pertaining to LF and HF form the upper and lower clusters, respectively. Overall, the changes in the PP displacement of the head are not related to the changes in the mean head AP position.

In Fig 4, the amplitudes of the PP displacement and AP positions of the head were plotted against each other during the LF and HF trials (the upper and lower clouds of data points, respectively). Each symbol corresponds to the mean value of both variables averaged cycle-by-cycle across participants. The data points were scattered in a moderate range, being overall displaced a little backwards with EO (open symbols), where the AP positions (abscissa) had negative values (compare with Fig 3A and 3B) and were broadly superimposed for the EO-TP and EO-TG conditions. These characteristics were not affected by the perturbation frequency. Instead, the PP displacement amplitudes (ordinate) were much larger at LF (top data cluster) than HF (bottom cluster), and largest with EC for both LF and HF. For all conditions, there was no consistent relationship between the mean PP and mean AP position (except for EO-TG and EC at LF, where the slope of the regression line was significant) (see Table 1). Overall, it appears that the contribution of the changes in the mean head position in space to the adaptation of the head PP displacements is of minor relevance.

**Table 1:** 

Head PP vs AP position		LF			HF	
	Regression Line	Value of ρ	p	Regression Line	Value of ρ	p
EO	y = -0.06x+9.2	0.026	0.89	y = 1.5x+5.5	0.125	0.53
EO-TP	y = 0.5x+10.2	0.319	0.10	y = 0.33x+4.3	-0.005	0.98
EO-TG	y = 1.4x+9.3	0.557	<0.01	y = -0.03x+3.9	-0.351	0.07
EC	y = -0.9x+10.8	-0.423	<0.05	y = 0.054x+5.8	-0.218	0.27

### Time-constant of the adaptation of head and pelvis PP displacements

The PP displacement amplitude of the head and of the pelvis increased progressively at LF for all visual conditions. Conversely, at HF, the PP amplitude of the head underwent a clear adaptation as well, but its values diminished over time. Adaptation was less evident for the pelvis at HF. For each participant and condition, the values of the amplitude of the head and pelvis PP displacements over the successive cycles were fitted with an exponential function and the time constant and adaptation index (AI) estimated. The goodness of the exponential fits was assessed by the Pearson’s coefficient (R). The pattern of adaptation was variable across the participants. R ranged between 0.31 and 0.64 for both head (significant in the 81% and 68% of the tests for LF and HF, respectively) and pelvis (significant in the 76% and 51% for LF and HF, respectively). In the following ANOVAs, we have included all the AI and time constant values, including those obtained from the fits with a non-significant R.

**Fig 5:**
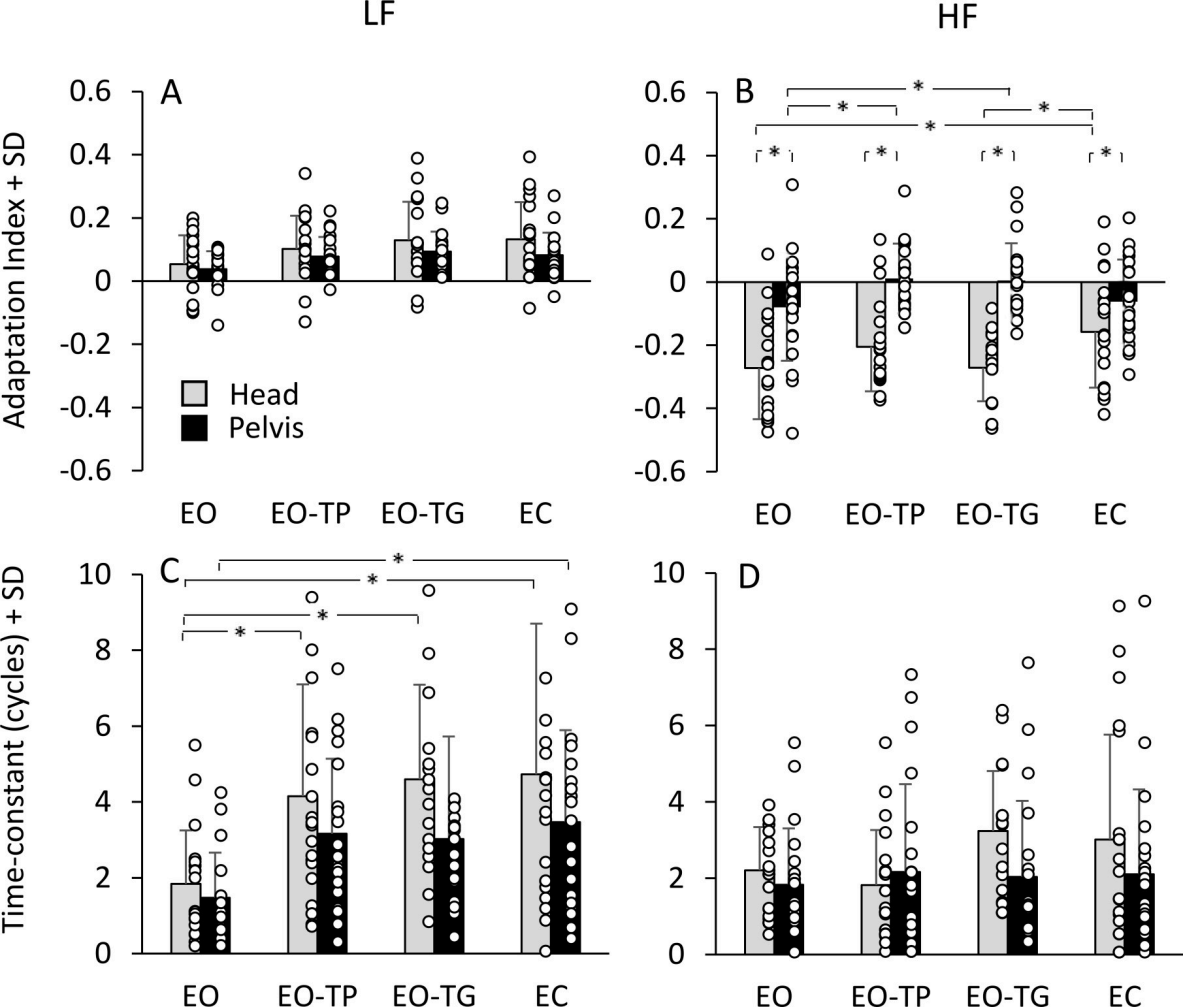
Parameters of the adaptation process. A and B: mean adaptation indices calculated for each visual condition under LF (A) and HF (B). Value of AI near zero indicate that the amount of adaptation is modest. This occurs when the value of the PP displacements at end of the translation cycles of the platform are similar to the PP displacement at the beginning of the platform translation. Positive values of AI indicate that the data showed an increasing exponential trend as a function of the subsequent translation cycles, while negative AI values were associated to a decreasing exponential trend. C and D: mean time-constants calculated by fitting an exponential curve to the PP displacement data over cycles for LF (C) and HF (D). Grey bars refer to head, black bars refer to pelvis. The white dots superimposed to the bars are the values of the AI and of the time-constant of each subject. Asterisks indicate significant difference (p < 0.05).

Fig 5A and 5B shows that the AI of the participants ranged between -0.5 and 0.4, where positive and negative values indicate a progressive increase and decrease in the amplitude of PP displacement, respectively. There was a large scatter of AI values across participants (open circles in A and B) and some adaptation indices were close to zero, indicating that adaptation was ineffective for some participants. Hence, we compared to zero the *mean* AI for each condition. The mean AI proved to be significantly different from zero for the head at both LF and HF (p < 0.001, for all visual conditions). The mean AI of the pelvis was significantly different from zero at LF for all visual conditions (p < 0.001) as well, but it was negative (barely so) or null at HF, indicating that the mean adaptation was of minor entity for the pelvis, absent when the supra-postural visual tasks were performed (p > 0.05, for both comparisons) and significant only for EO and EC (p < 0.05, for both comparisons).

After adjustment ([χ^2^(5) = 11.79, p = 0.038]; ε = 0.68 for the interaction effect between frequencies and visual condition), ANOVA on the AIs showed a difference between translation frequencies (main effect, F(1,19) = 88.7, p < 0.001) because the mean head PP displacement increased over the cycles at LF and decreased at HF. All frequencies and visual conditions collapsed, the head AI was larger than that of the pelvis (main effect, F(1,19) = 42.5, p < 0.001). The pelvis AI was small at both frequencies or almost null at HF as in EO-TP (LF, 0.078 ± 0.06; HF, 0.01 ± 0.11) and EO-TG (LF, 0.09 ± 0.06; HF, 0.005 ± 0.11). There was a significant difference between visual conditions (main effect, F(2.26,42.98) = 4.22, p < 0.05) and a significant interaction between visual conditions, translation frequencies and head and pelvis AIs (F(2.54,48.3) = 4.7, p < 0.01). This interaction effect depended mainly on the smaller AI of the pelvis, especially at HF and on the smaller AI of the head with EC at HF (-0.16 ± 0.18) compared to the other conditions (post-hoc, p < 0.01, for all comparisons).

The time constants of the progressive changes in the PP displacement ranged from almost 0 to 9 cycles for both head and pelvis and both frequencies. The values of the time constants are necessarily always positive, because they indicate a time-period elapsed from the onset of the exponential function (either increasing or decaying) (Fig 5C and 5D). After adjustment for the main effect of visual conditions (χ^2^(5) = 14.39, p = 0.013; ε = 0.68), a difference in the time constants was found between translation frequencies (main effect, F(1,19) = 8.76, p < 0.01), head and pelvis (main effect, F(1,19) = 15.59, p < 0.01) and visual conditions (main effect, F(2.05,38.99) = 6.03, p < 0.01). The mean time-constants were longer for LF (where adaptation featured a progressive increase in PP) than for HF and longer for the head than the pelvis. Thus, at HF, the adaptation process featured a rapid decrease compared to the slow increase at LF and the adaptation of the head was generally slower than that of the pelvis. There was a significant interaction between visual conditions and translation frequencies (F(2.58, 49.19) = 3, p < 0.05). At LF, the time-constants of the head with EO (1.84 ± 1.41 cycles, mean ± SD) were smaller than those calculated in all other visual conditions (post-hoc, p < 0.01 for all comparisons). For the pelvis, the time constant with EO (1.47 ± 1.2 cycles) was smaller than with EC (3.47 ± 2.4 cycles) (post-hoc, p < 0.05), but did not reach significance for EO-TP (3.16 ± 1.98 cycles) and EO-TG (3.02 ± 2.7 cycles) (post-hoc, p > 0.1 for both comparisons). There was no difference across the last three conditions (EO-TG, EO-TP and EC) for both head and pelvis (post-hoc, p > 0.2 for all comparisons). At HF, the time constants were not different across the visual conditions, for either head or pelvis (post-hoc, p > 0.4 for all comparisons).

### Head-pelvis coordination

**Fig 6:**
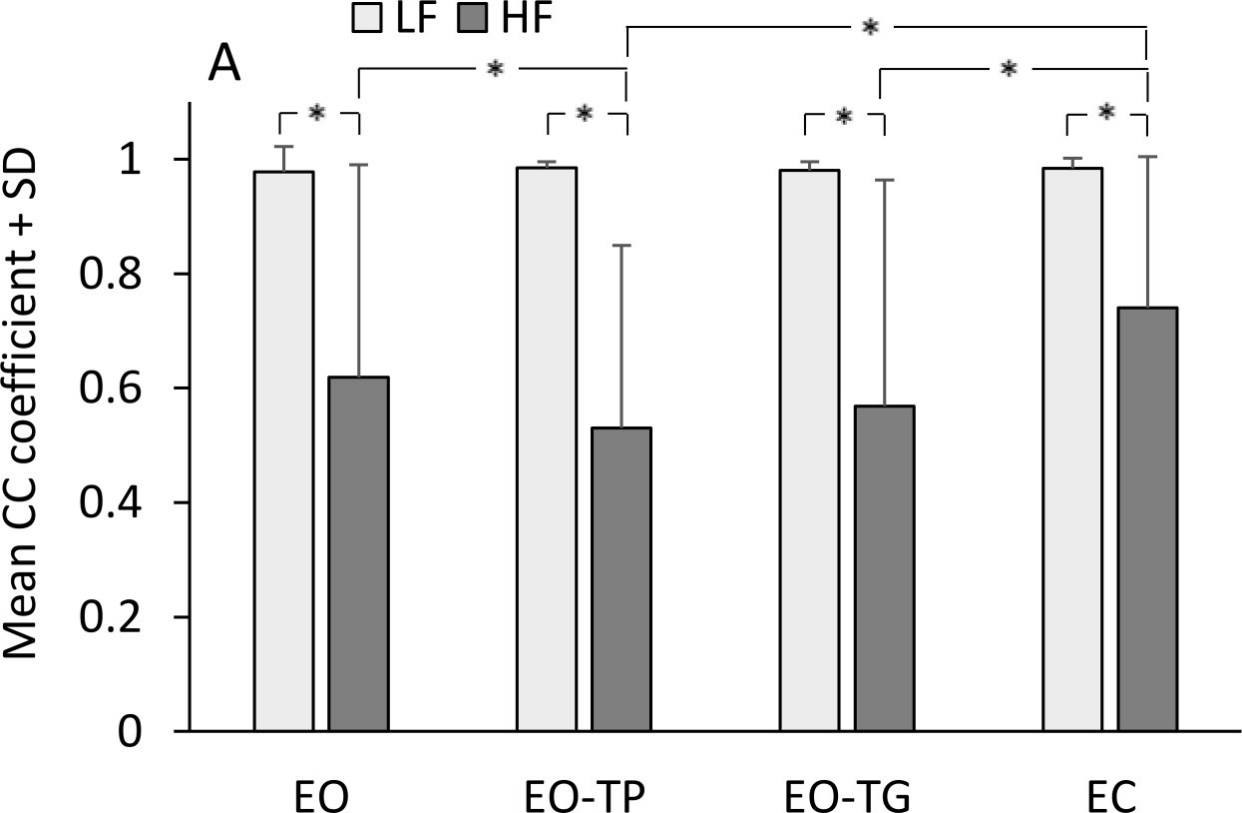
Mean CC coefficient between head and pelvis. Light grey bars correspond to LF, dark grey bars to HF. CC coefficients were always positive, indicating a synchronous displacement of head and pelvis. The CC coefficients are greater at LF than HF. Asterisks indicate significant differences (p < 0.05).

In order to understand whether the coordination between segments improved over time in parallel with the adaptation process, we estimated the values of the CC function of head and pelvis displacement for each participant cycle by cycle. In Fig 6, the mean values are reported. These values were always positive and much larger for LF than HF, indicating a better association between segment’s motion at LF. The difference in mean CC coefficient between LF and HF was significant (main effect, F(1,19) = 336.21, p < 0.001). There was a difference between visual conditions (χ^2^(5) = 36.91, p = 0.000001; ε = 0.56) (F(1.7,32.38) = 3.68, p < 0.05) and an interaction between frequencies and visual conditions (χ^2^(5) = 12.03, p = 0.034; ε = 0.71) (F(2.12,40.34) = 4.5, p < 0.05). In fact, visual conditions did not affect the coordination between head and pelvis at LF (post-hoc, p > 0.2 for all comparisons), while at HF, the smallest CC values were found with EO-TP and EO-TG (post-hoc, p < 0.05 for EC vs EO-TP and EO-TG and for EO-TP vs EO).

**Fig 7:**
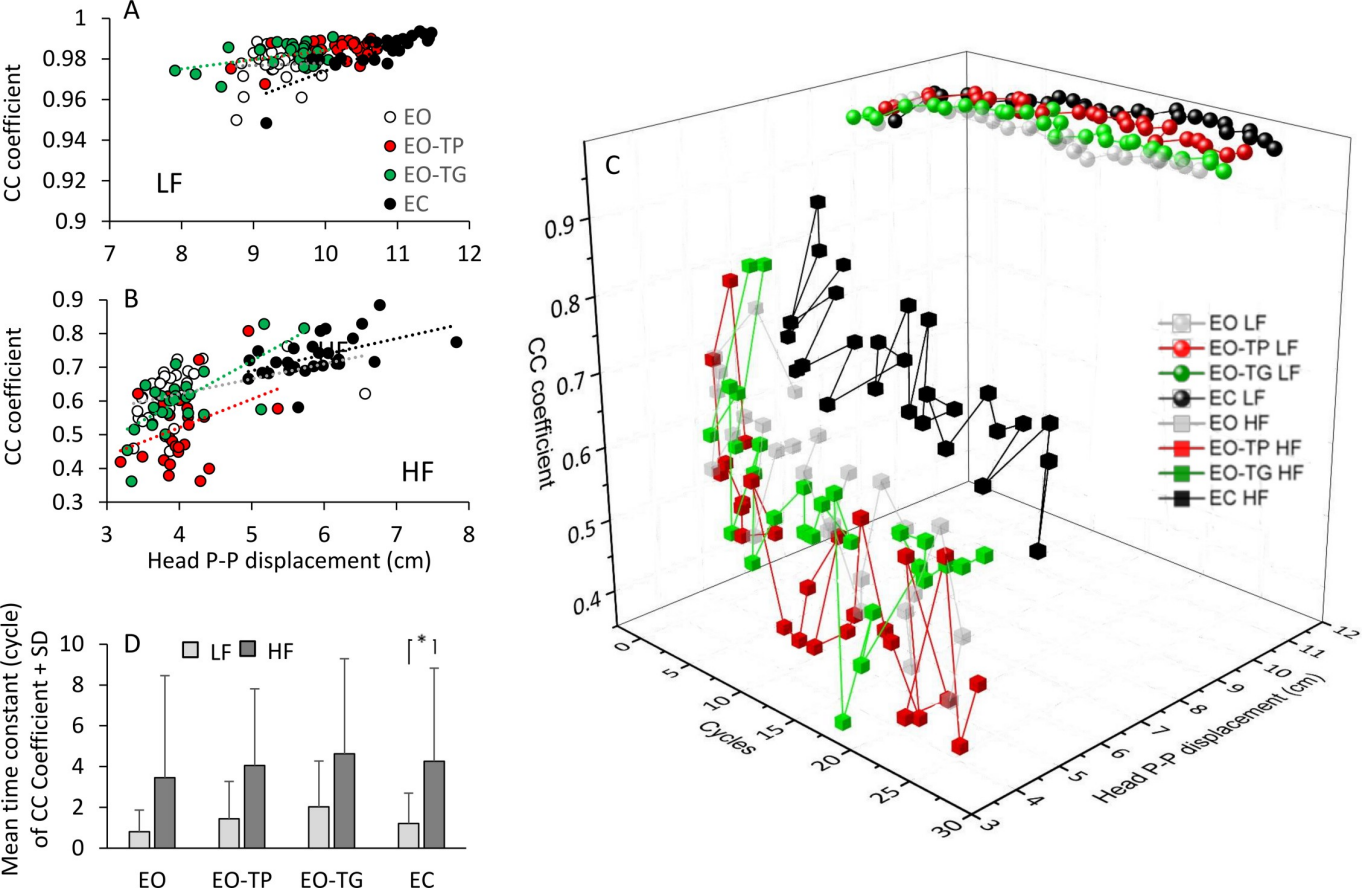
Head PP displacement and CC coefficient covary across cycles. A and B: The mean CC coefficient of each cycle was plotted against the corresponding value of the head PP displacement, for LF (A) and HF (B). White dots refer to EO, red dots to EO-TP, green dots to EO-TG and black dots to EC. C: the mean values of the CC coefficient and of the head PP displacement are plotted against each other and across cycles. Square symbols correspond to HF; circles correspond to LF. White symbols refer to EO, red symbols to EO-TP, green symbols to EO-TG and black symbols to EC. The panel D shows the mean time constant of the CC coefficients under the different conditions. Light grey and dark grey bars refer to LF and HF, respectively. The time constants are always smaller at LF than at HF. The asterisk indicates a significant difference (p < 0.05).

The relationships between the mean values of the CC coefficients of each cycle and the corresponding values of the head PP displacements are shown in Fig 7 for both LF (A) and HF conditions (B). High values of CC coefficient correspond to high values of PP head displacement. The regression lines are reported in Table 2.

**Table 2:** 

	LF			HF		
CC coefficient vs Head PP	Regression Line	R^2^	p	Regression Line	R^2^	p
EO	y = 0.0012x+0.97	0.001	0.85	y = 0.044x+0.44	0.13	0.06
EO-TP	y = 0.0044x+0.94	0.19	< 0.05	y = 0.084x+0.18	0.12	0.08
EO-TG	y = 0.0047x+0.94	0.19	< 0.05	y = 0.12x+0.13	0.49	< 0.001
EC	y = 0.013x+0.84	0.72	< 0.001	y = 0.05x+0.45	0.26	< 0.05

In Fig 7C, the mean values of the head PP amplitude and the mean values of the CC coefficient are plotted against each other as a function of the successive perturbation cycles. Obviously, at LF, both the head PP displacements and the CC coefficients moderately increased across cycles. The largest CC coefficient values and the largest PP amplitudes were observed in the adapted cycles (> 15th) of the EC condition. Instead, at HF, the head PP displacements and the CC coefficients decreased across cycles.

The head-pelvis CC coefficients varied progressively as a function of the successive cycles (Fig 7C) and were fitted with an exponential function. The goodness of the exponential fit was assessed by the Pearson’s coefficient. R ranged between 0.1 and 0.98 (significant in the 78% and 45% of the tests for LF and HF, respectively). Fig 7D shows the mean values of the time-constants of the CC coefficients for all visual conditions and perturbation frequencies. All the time constants calculated for the changes in the CC coefficients entered the following ANOVA. These values were different between frequencies (main effect, F(1,19) = 14.7, p < 0.01), smaller at LF (about 1–2 cycles) than at HF (about 4 cycles). There was no difference between visual conditions (main effect, F(3,57) = 0.85, p = 0.47) and no interaction between visual conditions and translation frequencies (F(3,57) = 0.05, p = 0.98). Therefore, the steady state in the coordination was reached sooner at LF (where increasing CC evolved along with the increasing head PP displacement) but later at HF (were the slow decreasing CC accompanied the head stabilization in space). Within each frequency, the different visual conditions did not affect the speed at which segments’ coordination reached a plateau.

We compared the time constants of the PP head displacement with those of the CC between segments. Interestingly, there was no overall difference between the time constants of the head PP displacement and those of the CC coefficient (main effect, F(1,19) = 1.44, p = 0.2). The time-constants tended to be longer at HF than LF, but the effect of translation frequency did not reach significance (main effect, F(1,19) = 3.26, p = 0.09). There was a significant interaction between frequency and time constant (χ^2^(5) = 11.6, p = 0.04; ε = 0.72) (F(1,19) = 22.8, p < 0.01), because the time-constants of the CC values were shorter at LF than HF, whereas the time constants of the PP displacements were longer at LF than HF (compare Fig 5C and 5D with Fig 7D). There was a difference between visual conditions (main effect, F(3,57) = 6.17, p < 0.01), because of the overall shorter time constants of both PP displacement and CC with EO than with the other conditions.

## Discussion

A fundamental property of the brain is its ability to adapt motor behaviour to changing bodily and environmental conditions. This provides the flexibility required to regulate the dynamics of movement and balance, set optimal body segment coordination and allow the expected accomplishment of a task. Several investigations have addressed the process of adaptation to repeated perturbations of balance [44, 46–48], and the effect of vision thereupon has been described [23, 24, 32, 44]. Adaptation implies a shift from the mostly reflexively mediated balance-correcting reactions to deliberate control of equilibrium, then again to automatic anticipatory postural activities [49], which are presumably less resource- and effort-demanding [32, 50, 51].

Adaptation to repeated perturbations of balance has been investigated [46, 52–58]. Vision of the environment is able to exert a substantial effect on head stabilisation when balancing on a continuously translating platform [8, 9]. This effect ensues very rapidly [23] and cannot be replaced by other senses [59]. While stable visual reference allows precise control of the balancing behaviour, balance without vision depends on proprioceptive and vestibular information [60, 61] probably at the expense of an enhanced cognitive effort [62]. Adaptation of the body motion to a complex supra-postural visual task may impose an additional load [63], as much as no-vision does. The extra-load would be related to the interaction of diverse continuously changing sensory inputs and to successful execution of the balancing movements with improved sharpness [64, 65]. The time constant of this adaptation would be comparable to, but certainly more protracted than that needed to incorporate an abruptly added (or withdrawn) tactile [19–21] or visual input [19, 66] when body posture is not challenged by continuous external perturbations. Adaptation seems to be preserved, though modified, in Parkinson´s disease [31] and across ages [55, 67], pointing to the wide-ranging relevance of research in this field for the understanding of balance control.

### Different adaptation patterns of head and pelvis motion under different visual conditions and platform translation frequencies

The present findings show that, under distinct visual conditions and translation frequencies, a steady state in head and pelvis oscillation is reached after few to several platform translation cycles. The period of adaptation that precedes steady state depends on vision and frequency conditions and is different for head and pelvis. Conversely, the body orientation in space (the mean AP position in space of head and pelvis) does not undergo any remarkable change over time. Apparently, individuals do not search for a more secure (protecting from a backward trip) but costly postural attitude (increase in postural muscle activity), such as for instance tilting forward in order to move the centre of pressure closer to the midpoint of foot length [68, 69]. Likely, the orientation in space normally adopted during quiet stance represents a strong reference around which perturbation-induced oscillations are organized [70], particularly so when the two supra-postural visual task are being performed.

Around the relatively unvarying mean position, the amplitude of the PP displacement varies over time. At low platform translation frequency (LF), the amplitude of the PP displacement of the head and pelvis is larger at steady state compared to the value at the beginning of the perturbation trials under the various visual conditions. The adaptation process features a gradual *increase* of the PP motion of the segments as a function of the successive cycles (the adaptation index has a positive value). The steady state is reached after one-two cycles with EO, while a slower time-course occurs in the other visual conditions, where the time-constant is not different among EO-TG, EO-TP and EC. Consistently, the amount of adaptation (the Adaptation Index, AI) was modest or negligible with EO for both head and pelvis, but large in the other visual conditions. Incidentally, it is appropriate here to consider that the values of the time constants computed from the exponential model, when applied to the EO data, may be affected by a bias connected with the small amplitude of the parameter B (the difference between intercept and asymptote of the fitted function). For small values of B, as occurs with EO where adaptation is quite limited, the variance of the estimated time-constant increases and its value becomes less reliable [71]. Hence, in spite of the significantly shorter time constant in the EO than the other visual conditions, the nominal value of the time constant with EO can be inaccurate.

The head motion in space depends on how the brain manipulates visual information according to the visual reference frames [34, 39]. During the adaptation period, vision of the text moving with the platform (EO-TP) produces stabilization of the head with reference to the target, rather than in space. As a consequence, at steady state, the head oscillates as much as the text when this is fixed to the moving platform, while it tends to keep a fixed position in space when the text is on firm ground [34]. Calibrating the feed-forward adjustment can require several cycles under these conditions compared to EO. A trial-and-error process may direct the time-consuming course leading to the slow increase in head PP displacement, dictated by the need for clear vision and easy reading. This may explain why the amplitude of the PP displacement is similar for EO and EO-TG [34], whereas the adaptation rate is shorter in the former than in the latter condition. With EC, the long time-constant of the adaptation of the PP head displacement is not different from that in the EO-TP and EO-TG conditions. These three conditions have in common an enhanced complication, connected with absence of vision (EC) or with the cognitive task (EO-TP, EO-TG). It appears that, although vision is of primary importance in the control of dynamic balance, equilibrium-control processes can modulate the impact of vision by taking into account critical constraints as to whether the supra-postural tasks may or may not require different head displacement amplitudes [72].

The magnitude and the direction of the adaptation process between the onset and the end of the perturbation sequence is strongly dependent on the frequency of perturbation. At HF, the PP displacements progressively *decreases*, particularly for the head (AI is negative). Interestingly, the decrease over time in the head PP amplitude at HF was more rapid than the increase at LF, suggesting a fast search for a steady state at the expense of the performance of the supra-postural task. It seems that, at HF, balance concern is the main driver of the balancing behaviour, as deduced from a gradually diminishing head PP motion in front of an almost constant mean PP amplitude of the pelvis. We should however note that the PP motion of the pelvis was variable across participants. The adaptation time course was also not significantly different across visual conditions at HF, even if there was a trend reminiscent of that observed at LF, suggesting again that the control of equilibrium overshadows the performance of the supra-postural visual task. Under a balance challenging condition (HF), the system for posture control likely disengages the command to the pelvis from that to the head in order to maintain the equilibrium while letting the head assist the visual task. Such process also underpins the EC condition, where the estimate of the gravitational vertical and head acceleration is driven by proprioception and vestibular input [73–75]. Probably, the variable pelvis control across participants is an indication of the attempt to draw a new strategy under unfamiliar challenging conditions. The small changes of the pelvis PP displacement from the beginning to the end of the perturbation period suggest that the balancing behaviour of the body on the translating platform is similar to a double-link pendulum [76], where the pelvis may not directly contribute to the changes of the head PP displacement amplitude. Rather, the minor displacements of the pelvis at HF compared to LF seems to represent a reference upon which the fine adjustments of head displacements occur. It has been noted already that minimization of the motion of the body’s centre of mass represents an important stabilization mechanism during continuous perturbations of stance [11, 46, 77]. Of course, these modest changes of the pelvis PP displacement across cycles could also affect the estimation of the time constants of the adaptation process. As for the head under EO condition at LF noted above, the estimation of the time constants of the changes of the PP amplitude are less reliable than for the cases in which the PP changes are more substantial.

### Coordination between head and pelvis AP displacements and their time constant

The CC between head and pelvis motion is very high at LF from the onset of the perturbation sequence and approaches one when the head PP displacement amplitude increases, indicating that a good coordination between head and pelvis is associated with ample head motion in the AP direction. The presence of an external focus of attention (the visual targets) has just a moderate effect on the CC during the adaptation period at LF. Oddly enough, the time constants observed for the adaptation of the CC coefficient are shorter than those for the adaptation of the head PP displacement amplitude at LF (about 1–2 cycles compared to about 2–4 cycles). Rapidly reaching a good association between head and pelvis motion seems to be a prerequisite for subsequent fine adjustment of the body AP displacement.

In the case of HF, contrary to LF, CC is small and decreases rapidly over time. Further, the time constants of the CC coefficient between head and pelvis are about 3–5 cycles compared to 2–3 cycles for the head PP displacement amplitude. A large change in the value of the CC coefficient between the centre of mass and the centre of foot pressure has been reported during adaptation at high frequency [78]. Here, the low CC values and the long time-constants of their changes are signs of a demanding effort to continually stabilize head displacement over the pelvis and in space (note in Fig 1 that the amplitude of the PP displacement of the head is being progressively reduced, while that of the pelvis remains remarkably constant). The adaptation time course of the CC is not different across all visual conditions within each frequency, suggesting that the performance of the supra-postural task does not interfere with the coordination between pelvis and head. The scarcely coordinated control of pelvis and head at HF [56] would require a costly and long process (a longer time constant than at LF) to maintain the equilibrium as if the performance of the visual tasks would be left to second plan compared to the control of balance. The difficulty in the balancing task and the need for swiftly minimizing energy use for balancing [79] seem to overwhelm the effect of the supra-postural task at HF.

The time constants of the exponential functions fitted to the changes in PP displacement and CC coefficients over time are located in a relatively restricted range of cycles, notwithstanding the largely different platform translation mode at the two frequencies. When the time-constants are expressed in seconds, they vary from about 5 s (EO) to about 20 s (head, EO-TP and EC) at LF, and from about 3 s (EO) to only about 6 s, at HF, therefore in a bigger range of time units (s) than the number of cycles. Therefore, we would suggest that the time course (but not the quality) of the adaptation process is partly set by the number of cycles and is based either or both on the sensory inputs originating during each cycle and on the anticipatory adjustments preceding the anterior and posterior dead points of each translating cycle. A broadly similar number of events has been counted in other balance adaptation studies [32, 47, 53, 55, 65, 80–83], including events of perturbed gait ([84], for a recent review). The main process of adaptation would be relatively independent from the time elapsing from the beginning of the sequence of perturbation but be calibrated on the frequency of discrete changes in the direction of the AP platform translations. If anything, when the perturbations have a higher frequency, the shorter time interval between the critical dynamic events would favour retention of the previous sensory information and facilitate the modulation of the successive automatic and anticipatory activities leading to adapted behaviour. This might partially explain the overall shorter time-constants of head PP adaptation amplitude (even when expressed in cycles) observed at HF compared with LF perturbations. Conversely, the longer time constants for the CC between head and pelvis motion at HF than LF would accompany the progressive loss of the coordination due to the primary need for stabilizing the head (and the upper body) in space. Another source for the slower changes in the CC at HF would be connected with the effort to attain a reduced number of synergies with practice, as it happens under high frequency conditions [85, 86].

### Exploiting vision at low and high platform translation frequencies

Full vision availability (EO, no task) undeniably confers a strong consistency and reproducibility to the cycle-to-cycle balancing behaviour at both LF and HF, so that little difference is observed between the early and the final translation cycles in the mean PP displacements of head and pelvis and the time constant of the adaptation is the shortest. This would mean that vision of the environment, a common condition during our everyday life, can send appropriate information about the body segments’ displacements in space. This information can calibrate both reflex and anticipatory activities within one-two perturbation cycles at the most [34]. The rapid modifications in head oscillation observed under EO are in keeping with the faster change in kinematics and EMG activity of the leg muscles observed some years ago in balancing individuals that abruptly passed from vision to no vision (compared to the opposite shift) [23] or in individuals voluntarily changing the balancing behaviour from one to another pattern [87].

At LF, a slower, progressive change in the body segments’ PP displacement amplitude occurs with EO-TP and EO-TG. This takes several cycles before the steady state is reached, much as in full absence of vision (EC). In the EO condition, individuals ‘ride’ the platform within a steady visual environment, where the visual cues are virtually static owing to the large distance between the head and the laboratory walls. In the EO-TG condition, the fixed target is part of the visual environment but there is a mismatch between the optic flow from the fixed target, relatively close to the body, and that from the environment. With EO-TP, the observer’s condition is similar to EO-TG before the start of the trial, but then the target motion in space (equal and synchronous to the motion of the platform) produces expansion and contraction of the optic flow from the peripheral visual field, while the changes in the optic flow from the central part of the field are minimized. Apparently, any central-peripheral mismatch in the optic flow is associated with a slower adaptation rate of the balancing behaviour. When performing supra-postural visual tasks, attending to the optic flow originating from the central retinal region [88, 89], more time would be necessary to integrate the input from this part of visual field and from the remaining sensory inputs (i.e., proprioceptive, vestibular) compared to EO. Perhaps, the two supra-postural tasks (EO-TP and EO-TG) and the EC condition rely on a similar motor integration process. It is not unlikely that the conscious effort of balancing with EC matches that of the supra-postural tasks. The head oscillations are large with EO-TP (text on the moving platform) [34], meaning that the contracting and expanding peripheral visual flow is not treated as a source of sensory input for head stabilization [10, 90, 91]. In spite of the different mean PP head and pelvis displacement amplitudes under EO-TP (large displacement), EO-TG (small, similar to EO) and EC (the largest) [34], the time-course of the adaptation process for the head was not different in the three conditions. Hence, we would only rely on two basic adaptation rates: a rapid one—driven by full-field vision availability, and a slower one depending on the processing of error signals driven by the control of distance from the visual target or by the proprioceptive and vestibular signals for the control of equilibrium (EC). The study [92] revealed that the availability of the dynamic visual cues is associated with considerable anticipatory activation of muscles producing large anticipatory CoP displacements. Possibly, with EO-TP, EO-TG and EC, the slow process of adaptation would be imposed by these unusual conditions implying a conflict between the stabilizing effect of vision and the new visual task opposing stabilization [79, 93].

The adaptation process at HF results in a remarkable rapid reduction in head PP oscillation, accompanied by a slow decline in the head-pelvis coordination. It is arguable that, at HF, reduction of oscillation is the crucial task of the adaptation process, aimed at minimizing the risk of falling, and that this prevails over other requirements related to the supra-postural visual task. The shift from head-on-trunk stabilization at LF to head-in-space stabilization at HF has been observed already [8, 9] and theoretically confirmed [94]. Moreover, it has been posited that direct visual feedback contributes only at low frequencies [95], because of the visual processing time that results in a long-latency time delay (> 200 ms). Here, the cycle duration at HF (0.58 Hz) is about 1700 ms, and the delay > 200 ms plus the integration time and the reweighting process [18–20, 64] might become critical for exploiting the visual input appropriately. Under different conditions, it was shown that for visual tilt stimuli individuals rely more on vestibular and proprioceptive information than on visual information [86]. This would contribute to selecting a ‘safer’ balancing behaviour by seeking a ‘head-fixed-in-space’ strategy [8]. Of note, under the three eyes-open conditions, pelvis PP displacements are smaller, and head oscillations even smaller, at HF than at LF, indicating that vision stabilizes the upper body similarly.

We note that the changes in PP displacements over time in a particular visual condition or those occurring across visual conditions took place unwittingly. Therefore, the motion pattern implicitly adopted by the balancing body would be the consequence of the visual feedback and of its progressive reweighting, rather than of an explicit prediction of the characteristics of the tasks. This time-consuming central process would be able to accommodate for the uniqueness of the sensory, motor, biomechanical, and supra-postural tasks, by playing upon the sensitivity of the reflex and anticipatory mechanism subserving the balancing behaviour. This study gives little hints as to the neural mechanisms responsible for the common adaptation rate under EO-TP, EO-TG and EC conditions. Vision modulates the contribution of vestibular noise, thereby reducing sway variability and allowing for lower sensory thresholds, in turn improving compensation [96, 97]. Vision recalibrates the proprioceptive neck input, and time is critical in this process [98]. Conversely, attention focused on a limited visual target with high salience would centrally gate the input from the peripheral vision [99] and entail a longer time period for reaching steady state in the balancing behaviour. Under different conditions, like standing on an unstable platform [100–102], a cognitive task enhanced automaticity of the balancing behaviour and allowed for reduced sway, but the time course of that effect was not identified.

## Limitations

There are limitations regarding the interpretation of the present results. The number of participants is limited, and motion of body segments and time constants of PP displacement amplitude and CC coefficient carry a considerable inter-individual variability. Moreover, the intra-individual variability in the oscillation pattern might attest the operation of confounding factors such as differences in reading strategies [103], weight attributed to expectation [104], apprehension [105] or implicit motor learning [106]. Individual drifts in antero-posterior position of head and pelvis, although cancelled in the population average, might have been a source of variability of the PP adaptation behaviour over time [107]. Actually, four of the participants did not exhibit the increase in head oscillation at LF and four did not show the decrease in head PP amplitude at HF. Paying more or less attention to the task or part of it could have influenced the balancing behaviour as well. This can happen during quiet stance [108, 109] and during continuous perturbations [110], and may be related to the ability to perform a motor task automatically. Moreover, the perturbations would have represented a more severe postural threat to some participants than to others [111], but this was not established. Likewise, the role of attention shift after the first perturbation cycle was not considered [112]. Our experimental approach does not allow to address all these issues, given the multifaceted patterns of brain areas activation following adaptation to postural perturbations [54], the presence of a condition of predictable continuous perturbations [54], and the amazing complexity of theories on this topic [14, 113–116]. As a consequence, our model used to calculate the time constant could have been too simplistic, or biased, in particular when the data variance around the population mean was high and the changes in amplitude of the steady state PP segments’ displacement were unimportant compared to the initial cycles.

The periodical platform translations were restricted to just two frequencies and one amplitude. This prevents a discussion on the effects of these constraints and narrows the possibility to draw a general notion on potential multiple adaptation modes. Further, the absolute entity of the adaptation was not as large as it had been described before [32]. This would be the consequence of the protocol. Here, participants were performing all the necessary trials at both frequencies within one day, the visual conditions (but not the frequencies) being mixed up randomly, in addition to having practiced a few preliminary cycles on the translating platform. Thus, participants were more than familiarized with the protocol, at both LF and HF. Further, two trials per condition were averaged to reduce variability. Therefore, the computed adaptation rates should have been little affected by the so-called `first trial effect´ [117, 118] that features a response contaminated by the startle reflex [66]. However, the trials in any particular visual condition were free from the trial-by-trial repetition effect because visual conditions were randomized. The frequencies were not randomized, in order not to mix up with the visual conditions the effects of the frequencies on the speed and extent of adaptation. In other words, what we see is the true adaptation to the supra-postural task, substantially deprived of the effects of the surprise or startle reaction [118]. The extent to which startle itself can interact and modulate adaptation to the supra-postural task cannot be evidenced here, but this effect cannot be totally excluded because the balancing behaviour accompanying the supra-postural tasks might be modulated by the habituating startle response [119].

Of note, the pelvis marker is just a substitute for the body’s centre of mass, particularly in a two-link pendulum model [120–124]. However, the remarkable consistency of the cycle-to-cycle position in space of the pelvis, compared to the ample changes in head PP displacement amplitude over time, allows to argue on the overall remarkable stability of the centre of mass of the balancing individuals, regardless of the perturbation frequency and visual conditions.

## Conclusions

The results support previous findings on the importance of vision in the control of vertical posture, provide evidence that the balancing behaviour, including the coordination between segments’ motion in continuously perturbed standing posture [110], is modified by the presence of different visual cues and perturbation frequency, and identify the time-period necessary for appropriate sensory integration to reach the final steady-state (see [125], for a synthetic update of recent investigations).

Continuous perturbations of stance have positive effects in the rehabilitation of balance in ageing [126], patients with neurological or vestibular deficits [127], cerebellar disease [128], stroke [83], Parkinson´s disease [6]. Exercises based on head movement have been beneficial in patients with vestibulopathy [129]. Prolonged exposure to optical flow during mechanical whole-body perturbations creates the conditions for modulating the integration of the proprioceptive, vestibular and visual inputs [58, 59, 130–132]. The present protocol might be valuable to further investigate the sensorimotor processes subserving calibration of dynamic balancing behaviour [133]. It could also serve to train and enhance diagnostic skills for elderlies [134] and Parkinsonian patients [135], who suffer from deficits in central processing of sensory information [136]. A recent review has considered balance priorities during complex balancing behaviours and suggested beneficial effects of dual-task training among elderly participants [137]. To date, investigations on the differences in adaptation to repeated perturbations between patients with Parkinson´s disease and matched non-disabled persons have shown analogies and differences [138–140]. Visuomotor adaptation to sudden perturbation of visual feedback of voluntary hand movements is abnormal in Parkinsonians [141, 142]. Shifts in vision conditions during continuous perturbations of balance do disclose abnormal delays in these patients [24]. The effects of perturbation frequency and of optic flow [143] and its interaction with other sensory inputs and supra-postural tasks on balance adaptation strategies have not been addressed in pathological states, yet [see 144, 145].

## Abbreviations

AP: antero-posterior
AI: adaptation index
CC: cross-correlation
EO: eyes open
EC: eyes closed
EO-TP: eyes open, reading a text moving with the platform
EO-TG: eyes open, reading a text fixed on firm ground
LF: low frequency
HF: high frequency
PP: peak-to-peak
